# Depression and Dementia in Older Adults: A Neuropsychological Review

**DOI:** 10.14336/AD.2021.0526

**Published:** 2021-12-01

**Authors:** Syuichi Tetsuka

**Affiliations:** Department of Neurology, International University of Health and Welfare Hospital, Nasushiobara, Tochigi, 329-2763, Japan.

**Keywords:** Alzheimer’s disease, dementia, depression, older adults, pseudodementia

## Abstract

Depression and dementia are the most common neuropsychiatric disorders in the older adult population. There are a certain number of depressed patients who visit outpatient clinics because they suspect dementia due to similarities in the clinical symptoms in both disorders. Depressive symptoms associated with dementia may be diagnosed with depression, and treatment with antidepressants is continued for a long time. Depression and dementia differ in their treatment approaches and subsequent courses, and it is necessary to carefully differentiate between the two in the clinical practice of dementia treatment. In this review, I describe the similarities between depression and dementia and how to differentiate depression in dementia treatment based on the differences and emphasize that there is a significant potential to cure depression, in contrast to dementia, for which there is currently no fundamental therapy. Therefore, it is important to recognize that depression and dementia may present with common symptoms and to appropriately differentiate depressed patients who are suspected of having dementia. Dementia is a disorder in which cognitive dysfunction is caused by a variety of causative diseases and conditions, resulting in impairment of activities of daily living. However, current medical science has had difficulty finding a cure for the causative disease. Based on clinical findings, it has also been shown that the degree of symptoms for preexisting psychiatric disorders is alleviated as the brain ages. In the presence of dementia, the speed of the alleviation will increase. The importance of focusing on the positive aspects of aging is also discussed.

In recent years, the number of healthy people in their 90s has been increasing in Japan. This is a phenomenon that did not exist two or three decades ago. A new definition has classified those aged 65-74 years as pre-old age, those aged 75-89 years as old age, and those aged 90 years or older as oldest-old age in Japan [1]. The number of oldest-old people is increasing to the point that a new classification is needed for people over 90. In super-aging societies, many people are now enjoying a long aging period. Many older adults enjoy hobbies and volunteer activities that they could not do when they were younger. When the various experiences of life are integrated in a relaxed manner, mental health will also be enriched. However, older adults are also exposed to mental health risks such as declines in physical and mental functions, health problems of their own and their families, death of close relatives, and loss of roles in work and society. The Japanese Ministry of Health, Labor and Welfare has reported an approximately 1.7 times increase in the number of outpatients with psychiatric disorders in Japan compared to 15 years ago (from approximately 223.9 million outpatients in 2002 to approximately 389.1 million outpatients in 2017). An upward trend has been noted in all age groups; however, it is particularly pronounced among outpatients aged 75 years and older, with an increase of approximately 3.2 times compared to 15 years ago [2]. In addition, there are a certain number of depressed patients who visit outpatient clinics on suspicion of dementia because depression and dementia in older adults show similarities in clinical symptoms. On the other hand, depressive conditions associated with dementia may be diagnosed with depression, and treatment with antidepressants may be continued for a long time. Depression and dementia differ in their treatment strategies and subsequent courses, and it is necessary to carefully differentiate between the two in dementia treatment.

This article presents a review of the effects of aging on mental health, how mental disorders change with age, and how medical care should respond to these changes in a super-aging society from the perspective of a neurologist specializing in dementia care for older adults.

## Aging effects on mental function

Aging is a phenomenon that cannot be avoided by anyone. The physiological functions of the heart, kidneys, lungs, and other vital organs peak in the thirties, and by the time we reach our sixties, the function of these organs is said to have declined to approximately 60% to 80% of their peak levels [3-5]. The brain is not an exception. In brain pathology studies, shortening and tortuosity of neuronal dendrites, reduction in spines, deposition of lipofuscin in neurons, and appearance of amyloid bodies are observed with aging [6]. Brain weight also peaks between the ages of 20 and 40 years and gradually decreases thereafter, with the rate of decrease slightly increasing after the age of 60 years [7].

What about brain function? The two types of intelligence suggested by Cattell, i.e., fluid intelligence and crystallized intelligence, are well known [8, 9]. Fluid intelligence is the ability to adapt to new situations, specifically the ability to calculate, memorize, think, and concentrate, and it is used to measure the intelligence quotient of an individual [10]. Crystallized intelligence is considered an intelligence that is based on fluid intelligence and is refined through education, culture, and experience [10]. Crystallized intelligence peaks around the age of 60 years, whereas fluid intelligence peaks around the age of 20-40 years and declines thereafter [11, 12].

Old age is also the period when dementia is most likely to occur. The frequency of dementia is said to be one in three to four people over 80 years of age and 70% of people over 90 years of age, and there has been a gradual shift in the perception of dementia in older adults as something that can affect anyone [13]. What is the impact of dementia on preexisting mental disorders? Most dementias are progressive degenerative diseases. As a result, the symptoms and features of psychiatric disorders, which are ultimately functional impairments, become less noticeable with dementia. In other words, the psychiatric symptoms that have persisted since adolescence and adulthood are gradually replaced by the symptoms of dementia caused by neuronal dropout, although they initially retain their vestiges. However, degenerative dementias, such as Alzheimer’s disease (AD), might actually cause or aggravate underlying neuropsychiatric symptoms during the early and middle stages of the disease, increasing the disability and disease burden of individuals already suffering from mental disorders.

## Mental health and depression in the older adult population

In old age, changes such as significant life events, decline in physical functions due to aging, and other factors can cause chronic stress, exposing older adults to mental health risks that may unknowingly progress to symptoms of depression. Symptoms of depression in older adults are often thought of as a general aging process, and the onset of depression can be noticed late [14-16]. A systematic review and meta-analysis of 24 articles published between 1999 and 2010 reported a prevalence of 7.2% for major depressive disorder (MDD) and 17.1% for depressive disorders in older adults over 75 years of age [17]. According to a report on the Center for Epidemiologic Studies Depression Scale (CES-D), a screening test for depression, the frequency of depression (score of 16 or higher) on the CES-D increased as age increased in both men and women. The frequency of depression (score of 16 or higher) increased as age increased, with 27.4% in the 75-80 age group, 33.3% in the 81-85 age group, 34.8% in the 86-90 age group, and 46.0% in the 91 years and older age group [18]. Considering that the incidence of dementia increases with age and that depression is frequently observed during the course of dementia, it is possible that pathological changes in dementia are associated with the development of depression.

The symptoms of depression in the older adult population are diverse, and depending on the symptoms that appear, the individual's feeling unwell and the impressions that the people around them have of them vary greatly. The main characteristics of geriatric depression are as follows: (1) Depressive mood and psychomotor retardation are unremarkable. (2) Anxiety and irritability are often prominent. (3) Many physical and psychological complaints characterize this condition. (4) Patients suffering from this condition are prone to complaints of poverty and hypochondriacal delusion. (5) It often causes sleep disturbance. (6) Suicide attempts by patients with this condition are frequent. (7) This condition is prone to recurrence and has a poor prognosis [14, 19-23]. MDD in the Diagnostic and Statistical Manual of Mental Disorders-IV (DSM-IV) is characterized by the presence of at least one of the following symptoms: decreased interest and pleasure and depressive mood. However, depressive mood tends not to be present in older adults with depression, and a decrease in interest and pleasure seems to be the main symptom; thus, the DSM-IV criteria based on their emphasis on depressive mood may underestimate depression in older adults [24, 25]. This feature is extremely important in supporting older people with depression. In other words, if we focus on the "depressive" appearance, we will miss the depressed older adults who need support. Clinicians should be well aware that many depressed individuals in the community do not exhibit the depression or depressive mood observed among severely depressed patients who visit psychiatric hospitals. Even severely depressed patients may not exhibit depressive mood, and even psychiatrists cannot diagnose depression just by looking at the patient. Depressive mood is a symptom that is difficult to grasp even by specialists. To identify depressed individuals, it is helpful to look for a decrease in interest and pleasure, which is a main characteristic of depression in older adults [26]. This symptom can be identified by paying attention to the changes in the lives of older adults. When asked “Have you ever lost enjoyment of things you used to enjoy or lost interest in activities you used to be interested in?,” those with this symptom answered “Recently, I do not enjoy walking in the morning,” “I went hiking with my friends, but I did not enjoy it at all, unlike before,” or “I used to enjoy taking pictures with my favorite cameras when I go out, but I have not taken any photos in the past 2 years. I have lost attachment to my favorite cameras.” In this way, patients inform about their lifestyle changes to clinicians. These changes have been ongoing for several years. Because the diagnostic criteria for MDD require persistence of symptoms for 2 weeks, asking only about recent changes will result in an answer like “There is no particular change.” The key to interviewing is to ask about changes over a more extended period. It is noted that depression in older adults is referred to as “depression without sadness,” and that depressed older adults often exhibit irritability and withdrawal rather than mood discomfort [27].

Depressed older adults with decreased interest and pleasure are aware of the changes that are happening to them and find these changes troubling. It is not uncommon for patients to self-criticize the limitations in their lives based on a decrease in interest and pleasure, saying that they have become lazy or pathetic. In these respects, the loss of interest and pleasure in depression differs from the apathy that occurs in dementia. Older patients with apathy do not feel troubled by the fact that they no longer perform household chores or hobbies. The decrease in interest and pleasure caused by depression can be improved by treatment. Unlike the apathy with dementia, depression is subject to practical secondary prevention. In identifying depression, it is important not to overlook changes in life, and particular attention should be paid to bereavement as it causes the onset of MDD in later life [28]. Depression in older adults is not limited to a leave of absence from work but can also lead to a complete withdrawal from work, such as retirement from the Silver Human Resource Center program or the closing of a self-employing business. Even for unemployed individuals, a reduction in lifestyle, such as a decrease in hobbies and household activities, leads to a decline in muscle strength in the legs and feet, which leads to a decrease in activities of daily living (ADLs). Early identification of depression is a practical means of preventing the decline in ADLs. A recent systematic review has reported that depression was the most important risk factor associated with suicidal behavior in later life and suicidal behavior in older adults was strongly associated with physical illness and disability; moreover, stressors, living alone, and poor health were also predictors of suicide attempts [29]. In particular, older adults who experience social isolation are found to be at higher risk of increased morbidity, decreased immune function, depression, and cognitive decline [30]. Loneliness, especially in later life, is closely associated with depressive mood, decreased happiness, and even increased mortality [31]. Older patients may not immediately recognize that any of these symptoms are associated with depression. Therefore, clinicians need to carefully consider the relevance of these symptoms in terms of assessing older patients [32].

## Depression mixed with dementia

The percentage of patients with dementia who meet the diagnostic criteria for a depressive episode has been reported to be 33.2% and 43.7% for AD and Lewy bodies (DLB), respectively, 60% for vascular dementia (VaD), and 33% for frontotemporal lobar degeneration (FTD) [33-35]. It has been reported that 19.7% of DLB patients meet the diagnostic criteria for MDD in the DSM-IV, which is higher than the 8.7% of AD patients [33]. A total of 20,892 patients from 57 studies published between 2002 and 2015 were included in the analysis, and the comorbidity of depression in patients with mild cognitive impairment (MCI) was reported to be 32% [36]. In addition, dementia causes changes in daily life due to memory impairments and other cognitive and life-functioning impairments. In particular, in the early stages of the disease, the patient is often aware and conscious of these changes, which can contribute to anxiety and depression, but there is no relationship between the progression of dementia and the frequency of depression and depressive state. In other words, dementia is a condition in which the gap between one's own intentions, images and reality becomes large due to a decline in ability and function, regardless of the stage of the disease, and the patient suffers from stress on a daily basis. It is thought that reactive depressive state is often present, and this is an important background for understanding depressive symptoms such as behavioral and psychological symptom of dementia (BPSD) [35]. It has been reported that apathy in AD involves neuronal loss, neurofibrillary changes and white matter hyperintensities in regions considered important in frontal lobe-subcortical circuitry (anterior cingulate gyrus, basal ganglia, hippocampus and medial frontal gyrus) [37]. The results of functional imaging studies have also shown a decrease in blood flow in areas included in the subcortical network of the frontal lobe [38]. On the other hand, impairments in the frontal-striatal-subcortical-limbic circuitry is thought to play an important role in depression in AD [39]. According to this network view, the diversity of apathy symptoms in AD may reflect differences in the many structures and circuits that are impaired, and the comorbidity of apathy and depression may be related to the fact that they share common neural circuits.

Regarding depression as a prodromal state of dementia, Ownby et al. conducted a meta-analysis of 9 case-control and 11 cohort studies and reported odds ratios of 2.03 and 1.90 for the development of AD in patients with a history of depression compared with those without a history of depression, respectively [40]. Other meta-analyses have reported odds ratios of 1.65 and 2.52 for the development of AD and VaD, respectively, in older patients with a history of depression compared with patients without a history of depression [41]. In addition, a systematic review of longitudinal studies of pseudodementia (otherwise known as depression-related cognitive dysfunction) reported that 33% of patients with depressive pseudodementia had progressed to irreversible dementia at follow-up [42]. Recently, depression has received special attention as one of the precursors of DLB, and a retrospective study of 90 patients with DLB reported that depressive symptoms preceded memory impairment by an average of 4.8 years [43].

## The relationship between depression and dementia

**Figure 1.**
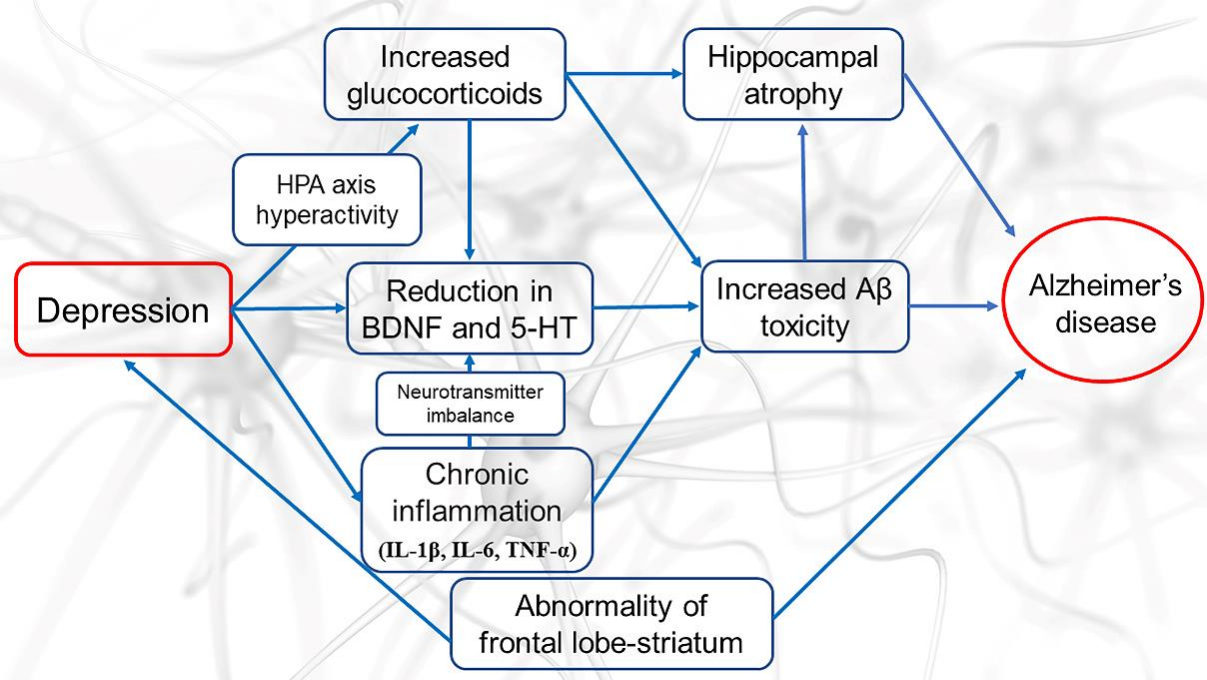
**The possible mechanisms leading from depression to the development of Alzheimer's disease (AD).** Hypothalamic-pituitary-adrenal dysfunction, decreased neurotrophic factors, and chronic inflammation play a central role in the pathogenesis by which depression causes AD. Increased glucocorticoids and proinflammatory cytokines and decreased brain-derived neurotrophic factor (BDNF) and 5-hydroxytryptamine (5-HT) lead to high beta-amyloid toxicity, which first causes hippocampal atrophy. As a result, the progression from depression to AD is facilitated. HPA; hypothalamic-pituitary-adrenal.

Although we have already mentioned that apathy in dementia is different from the symptoms of depression, it is well known that depression appears in the course of dementia. Some patients with dementia become indistinguishable from depressed patients and require antidepressants, while others have underlying depressive symptoms but forget that they sometimes complain of depression due to poor memory. Thus, the presentation of depression in dementia varies widely. On the other hand, epidemiological studies have shown that depression and depressive symptoms are early symptoms or risk factors for dementia [44]. A retrospective cohort study has been recently reported using healthcare data from 2012 (baseline) and 2016 (follow-up) for older adults (75 years or older) in Japan. The results showed that 226,738 older adults who had not been diagnosed with dementia at baseline were followed up, and 26,092 older adults were diagnosed with dementia after adjusting for confounding factors. Depression was also identified as a factor that was significantly associated with subsequent dementia diagnosis (odds ratio: 1.38, 95% confidence interval: 1.31-1.44) [45]. The results of a meta-analysis of 23 regional cohort studies in people aged 50 years and older also showed that a history of depression is a risk factor for dementia, AD, and vascular dementia [41]. Longitudinal studies have confirmed that there is a graded association between the severity of depressive symptoms and the risk of dementia, with the risk being more pronounced in severe depression [46]. In addition, a history of depression long before the onset of dementia has been reported to be a risk factor for developing dementia [47]. Studies have shown a strong association between the number of depressive episodes and the risk of developing dementia, with each additional episode of depression increasing the risk of dementia by 14% [48, 49]. A comparative study of serum Amyloid-β (Aβ) levels in a group of patients diagnosed with MDD by the DSM-IV and healthy controls reported that the MDD group had a significantly higher Aβ40/Aβ42 ratio than the control group [50]. In addition, significant Aβ deposition in the lateral temporal lobe and posterior cingulate gyrus has been reported in geriatric depressed patients compared to healthy older adults [51]. It is widely known that hippocampal atrophy is an important finding in AD, and it has been reported that hippocampal volume is reduced in depressed patients compared with normal subjects [52]. Since the accumulation of Aβ in the brains of AD patients begins more than 20 years before the onset of dementia symptoms, the pathological changes in the brain caused by early AD may affect the onset of depression. In addition, the concept of amyloid-associated depression, which suggests that there is a type of geriatric depression that is affected by Aβ and that depression exists as a precursor to AD, has been supported [53]. Several possible mechanisms by which depression may be a risk factor for the development of AD have been postulated in terms of the neurodegenerative effects of depression (Fig. 1) [44, 54]. Patients with depression have a high incidence of hypothalamic-pituitary-adrenal (HPA) axis hyper-activity and higher levels of glucocorticoids [55]. It has been reported that elevated levels of glucocorticoids cause accelerated hippocampal atrophy and enhanced Aβ toxicity [56, 57]. Elevated levels of glucocorticoids also induce a reduction in brain-derived neurotrophic factor (BDNF), a decrease in neurogenesis, and an increase in apoptosis [58]. In a significant proportion of patients with depression, chronic mild inflammation is present, and a number of studies have reported increased circulating peripheral and central proinflammatory cytokines (IL-1β, IL-6, TNF-α), inflammatory mediators, and acute-phase proteins (CRP) in patients with depression [59-62]. Mild chronic inflammation has been shown to reduce 5-hydroxytryptamine (5-HT) synthesis in brainstem nuclei and decrease synaptic availability [63-65] (Fig. 1). This can lead to an inadequate supply of monoaminergic neurotransmitters to cortical areas (neurotransmitter imbalance), which then represents a fundamental mechanism in the pathophysiology of depression [66]. Because BDNF and 5-HT, which is reduced by depression, is an important protein involved in neuroplasticity and hippocampal neurogenesis, low serum BDNF and 5-HT levels have been reported to be a risk factor for AD [67, 68]. Thus, a vicious cycle has been postulated in which increased cortisol levels due to depression lead to decreased BDNF function, and pathological degeneration of AD leads to further decreased BDNF function (Fig. 1). Due to these pathological mechanisms, in patients suffering from depression, there may be more cases of impaired thinking, judgment, and cognitive function. Although rare, there are cases of depression that develop into severe dementia-like conditions. When an older adult is depressed and cognitive function is impaired, it should not be so quickly judged as an early symptom of dementia. Treatment of depression can improve cognitive function [69].

Depression is a condition in which symptoms centering on depressive mood and loss of interest or pleasure are prolonged over a period of weeks and accompanied by a number of psychological symptoms such as depression, inactivity, anxiety, loss of appetite, sleep disturbances, agitation, and fatigue. Many patients complain of difficulties thinking, concentrating, and making decisions and often have difficulty performing tasks that require a high level of cognitive function. Especially in older adults, memory impairment is often the main complaint and is often mistaken for an early symptom of dementia. In fact, recent studies have shown that the percentage of depressed patients with cognitive impairment that meets the diagnostic criteria for MCI is 48% to 52%, accounting for approximately half of all depressed patients [70-72]. As mentioned in the previous paragraph, such cognitive dysfunction associated with depression was called “pseudodementia” for a long time, and the treatment of depression, which is the cause of pseudodementia, reversibly improves cognitive dysfunction [73]. In short, pseudodementia is a psychiatric disorder disguised as a neurodegenerative disease. In this multicenter study, the frequency and severity of each BPSD were evaluated in four major dementias: AD, dementia with DLB, VaD, and FTD, and depression and anxiety were observed in more than 20% of patients with very mild or mild dementia; in particular, the severity of depression and anxiety was stronger in patients with very mild or mild DLB [74]. More than half of the patients with any of the four major dementias showed abulia and indifference, regardless of the severity of the disease. These results indicated that depression is sometimes accompanied by cognitive dysfunction, while dementia often presents depression, abulia, and anxiety as BPSD from the beginning and that depression and dementia present similar clinical symptoms. One population-based study that recruited patients from primary care medical practices reported that 0.6% of older adults over 65 years old had depressive pseudodementia [75]. Thus, pseudodementia is a major burden in society, considering its prevalence and the development of serious cognitive and functional impairments. During the 1980's and 1990's, there was a great deal of academic activity on pseudodementia, but this interest seems to have declined slightly in recent years [76]. However, this condition should not be taken lightly, as it places a heavy burden on the patient and increases the risk of dementia.

Mild behavioral impairment (MBI) is a newly recognized neurobehavioral syndrome characterized by the emergence and persistence of diverse neuropsychiatric symptoms for more than 6 months later in life, which include behavioral changes and mild psychiatric symptoms, especially disinhibition and depression [77, 78]. This syndrome is comprised of neuropsychiatric or behavioral symptoms as a precursor to cognitive decline and dementia. Further, this syndrome has already been recognized in the community and in clinical research cases and has been shown to be present not only in many older patients with sustained neuropsychiatric symptoms or MCI but also in some cognitively normal older adults [79-82]. A recent a systematic review and meta-analysis based on 11 studies including 15,689 subjects (older adults) has showed that the pooled prevalence of MBI was 33.5% (95% confidence interval: 22.6%-46.6%) [83]. Additionally, 18.2% of the older adults without dementia were found to have MBI [84]. MBI might present significant diagnostic challenges, as it may mimic many symptoms of depression in older adults that are actually a result of underlying AD pathology (Aβ) [85]. To compare the risk of progression to dementia, an observational and analytic study was conducted among patients with MBI, MCI, and a psychiatry group; as a result, the rate of transition to dementia was determined to be significantly higher in MBI (71.5%), followed by the MCI-MBI overlap (59.6%) and MCI (37.8%) groups, compared to psychiatry group (13.9%) (Log-rank *p* < 0.001); further, the study indicated that MBI might be a clinical predictor for cognitive decline and dementia [86]. In addition, some studies demonstrated that the emergence of sustained depressive symptoms increases the risk of dementia, and depression is more likely to be indicative of dementia in the early stages [87-89]. Despite the increasing evidence of an association between depression and dementia, the neuropathological mechanisms of depressive symptoms, which are predictive of the onset of dementia, have not yet been reliably elucidated [89]. However, as mentioned above, there are several possible mechanisms by which depression may be a risk factor for the development of AD, in terms of the neurodegenerative effects of depression (Fig. 1). In a further recent study, it was found that MBI is associated not only with Aβ but also with tau pathology in older adults [85, 90]. Furthermore, detailed study of the symptom domains of emotion dysregulation using the MBI framework is important not only to improve our understanding of neuropathology but also to develop prevention and treatment strategies for those at risk. In summary, as shown below, the link between depression and dementia continues to be explored in search of the underlying reasons and the direction of the causal relationship.

- Depression complicates the dementia condition of AD.
- Depression as a risk factor for AD
- Depression, as one of the MBIs, might be a clinical predictor for cognitive decline and dementia.

Although there is no supporting evidence yet, these associations suggest that depression can be a potential modifier of dementia development and that the treatment of depression may slow down the progression of dementia [66].

## Differentiation between dementia and depression

Because of these similarities, depressed patients are often suspected of having dementia and are referred to dementia specialists. Therefore, it is important to understand the differences that should be considered in differentiating depression from dementia. Depression associated with AD is more variable and less persistent than depression, with irritability and anxiety being more prominent than sadness [91]. In addition, it was reported that psychomotor retardation was significantly higher, and suicidality, appetite loss, and weight loss were significantly lower based on Hamilton Depression Rating Scale scores in depression associated with AD [92]. On the other hand, cognitive dysfunction is mainly an attention disturbance in depressive pseudodementia, whereas in AD, recent memory impairment is remarkable from the beginning. At the time of examination, depression is marked by a tendency to give up effort in response to questions and to become serious and sometimes exaggerated about forgetfulness, while AD is accompanied by saving appearance responses, head-turning signs, and a lack of insight into memory loss. Apathy is a common symptom of BPSD, but it is often difficult to distinguish apathy from depressive state. However, apathy is mainly characterized by a lack of interest and illness awareness and is closely related to cognitive dysfunction. Depressive state is mainly characterized by depressive mood, suicidal thoughts, and self-blame and often causes distress to the patient and caregivers. There is a significant difference in the characteristics of the two conditions, suggesting that they are based on different neural substrates. In clinical practice, there are many cases in which apathy is accompanied with depression or depressive state, and it is important to differentiate between the two and to treat and respond to each appropriately. The neuropsychiatric inventory (NPI) is a rating scale for BPSD, and the 12 symptoms are categorized into four factors: hyperactivity (irritability, agitation, euphoria, disinhibition, aberrant motor behaviour), psychosis (delusions, hallucinations, night-time behaviour disturbances), affective (depression, anxiety), and apathy (apathy, night-time behaviour disturbances, appetite and eating abnormalities). Furthermore, apathy and depression can be evaluated as separate factors [93]. However, in the NPI, apathy is described as "decreased interest in surroundings and things, and decreased motivation to start new things," which is seen as a symptom similar to decreased motivation and interest in depression, making it difficult to differentiate between depression and apathy [93]. Thus, it is difficult to distinguish apathy from depression by using only a rating scale, and an interview with the patient is important in clinical practice. In both cases, there is a similar decrease in activity and loss of motivation and interest, but when accompanied by sadness and subjective changes in sleep and appetite, there is a possibility of depression requiring treatment, and proactive treatment is necessary [94]. In terms of the course of the disease, cognitive dysfunction in AD progresses slowly, whereas cognitive dysfunction caused by depression, which is referred to as pseudodementia, tends to have a clear onset and rapid deterioration [95]. Depression is a subacute symptom change, and the appearance and persistence of depressive mood, psychomotor retardation, decreased interest and pleasure, decreased appetite, and sleep disturbances are often observed with symptom awareness and distress conflicts. On the other hand, dementia-derived symptoms, or BPSD, include chronic cognitive dysfunction, depression, anxiety, and irritability, but they are situation-dependent and characterized by significant fluctuations in symptoms in relation to time, place, and person. Appetite is often maintained, and there is little awareness of sleep disturbances. The patient's thoughts and movements are slow, but he or she may enjoy spending time in an appropriate environment. From these symptoms, it is necessary to differentiate between depression based on biological factors and depressive symptoms as an emotional response (BPSD) and to consider treatments and responses for each. Cognitive function screening tests, such as the Mini-Mental State Examination, are useful in differentiating pseudodementia from AD, depending on the type of test items that show a decline in scores and the attitude toward taking the test [96]. Pseudodementia tends to be associated with a decline in verbal fluency rather than a decline in delayed recall, which is different from AD patients who tend to lose points in delayed replay. However, this test has several limitations [ceiling effect, limited specificity, and confounder sensitivity (sociodemographic characteristics, sensory impairment)] that make it not suitable for a comprehensive cognitive function assessment. Instead, for a thorough evaluation of cognitive performance and assessment of individual cognitive domains (memory, language, executive functioning, attention speed, visuoperceptual ability), more appropriate tools, such as comprehensive neuropsychological test batteries, should be deployed. The use of comprehensive neuro-psychological test batteries for cognitive function assessment not only improves the accuracy of dementia (and type thereof) diagnosis but also helps differentiate between dementia and other causes of cognitive impairment (such as depression) based on the pattern and type of cognitive domains affected [97]. The clock drawing test is also useful in assessing constructive ability, which is not abnormal in depression but often impaired in AD [98]. In AD, specific findings such as atrophy of the temporal and parietal lobes on brain morphology images, such as brain MRI, reflective of neurodegenerative diseases, decreased glucose metabolism in the temporal and parietal lobes on functional brain images, increased total tau and phosphorylated tau in cerebrospinal fluid, positive findings on amyloid PET scan reflecting cerebral Aβ accumulation, and decreased Aβ42 in cerebrospinal fluid, are useful for diagnosis [99].

On the other hand, it is not difficult to differentiate DLB from depression when specific symptoms such as parkinsonism, REM sleep behavior disorder, and visual hallucinations occur, but it is often difficult to differentiate DLB from depression when these symptoms do not occur. Depression is more frequent in DLB than in AD [33]. DLB often presents with more severe depression than AD, and the prevalence of abulia, apathy, and anxiety is also higher [33]. In the early stages of cognitive dysfunction, recent memory impairment is not noticeable, and attention disturbance and visuospatial deficits are more prominent. Therefore, it is difficult to distinguish DLB from depressive pseudodementia based on only depressive symptoms and cognitive dysfunction [100]. Autonomic disturbances such as constipation and orthostatic hypotension, which are supportive features of DLB, as well as hypersensitivity to antipsychotics, are not highly specific, when considering depressed patients and older adults in general [100]. In addition, it is now known that many cases of DLB are initially diagnosed with MDD. Although it is good that the prodromal state of DLB is now actively included in the differential diagnosis of depression in older adults, it is possible that DLB is being overdiagnosed because of this trend. If the diagnosis of depression is made but the presence of supportive DLB’s features strongly suggests the possibility of a prodromal state of DLB, follow-up may be required, including dopamine transporter scintigraphy, MIBG myocardial scintigraphy, and polysomnography, which are index biomarkers of DLB, as well as confirmation of response to treatment [100].

**Table 1:** Differentiating depressive pseudodementia from dementia.

	Depressive pseudodementia	Dementia
Response and attitude toward functional decline	Attitudes that overestimate and pessimistically emphasize the decline in abilities	Lack of interest in or denial of diminished capacity, or an attitude of mending
Type of onset	Onset time can be identified on a weekly to monthly basis.	Slow onset; onset time only identifiable in seasons or years.
Variability and environmental reactivity	Diurnal variation that worsens in the morning; constancy even when the environment changes	Variations in attention and concentration suggest DLB; mood and motivation improve with positive environment and interpersonal interaction.
Psychiatric symptoms	Feelings of sadness and remorse are present that are sometimes accompanied by feelings of hopelessness and thoughts of death, and sometimes accompanied by psychic delusions, delusions of guilt, and delusions of poverty; in rare cases, delusions of nihilism and immortality may accompany the symptoms.	Lack of sadness and remorse, lethargy, and indifference (apathy) are the main symptoms. Emotional incontinence suggests VaD, sometimes accompanied by delusions of being robbed; repeated and specific visual hallucinations suggest DLB, mixed apathy and homophobic behavior suggest FTD.
Movement symptoms	Not acceptable.	Parkinsonism and easy falling suggest DLB and VaD, and hemiplegia and dysarthria suggest VaD.
Sleep	Wakes up in the morning; going to sleep is an obstacle.	Gradual rhythm disturbance, day and night reversal; REM sleep behavior disorder suggests DLB.
Appetite, weight	Decreased appetite, sometimes increased appetite, with weight changes on a weekly to monthly basis.	Slow weight loss: anorexia, overeating, and rapid weight gain suggest FTD.
Simple cognitive function test findings	Answers such as "I don't know" or "I don't remember" and slow thinking. Careless mistakes in continuous subtraction and reverse chanting; delayed playback is impaired, but reaffirmation is maintained; figure sketching and drawing will be maintained.	Wrong answers and mending, and turning around signs. Thought laziness and walking away behavior suggest FTD; markedly delayed playback and impaired reification suggest AD; impairments in figure copying and drawing suggest AD and DLB.
Morphological brain imaging	Normal or age-related changes, mild atrophy of the hippocampus, olfactory cortex, amygdala, and frontal lobe, and mild deep white matter ischemic changes.	Atrophy of the hippocampus and olfactory cortex and parietal lobe suggests AD; medium to large infarcts, multiple infarcts, infarcts at strategic sites, and high levels of white matter lesions are indicative of VaD; severe frontal and temporal lobe atrophy suggest FTD.
Functional brain imaging (decreased cerebral blood flow and metabolism)	Normal or mildly impaired frontal lobe function.	Functional decline in the posterior cingulate gyrus and anterior portion of the scapula suggests AD; functional decline in the occipital lobe suggests DLB; a high degree of frontal and temporal lobe dysfunction suggests FTD.
Other functional imaging tests	Dopamine transporter and MIBG myocardial scintigraphy are normal.	Decreased dopamine transporter uptake in the basal ganglia or decreased uptake on MIBG myocardial scintigraphy suggests DLB.

AD; Alzheimer's disease, VaD; vascular dementia, DLB; dementia with Lewy bodies, FTD; frontotemporal dementia

It is important to differentiate depression in the clinical setting of dementia treatment, taking into account the abovementioned differential points. Table 1 summarizes the key points for the differential diagnosis of depressive pseudodementia and dementia in current clinical practice. However, there are cases in which correct diagnosis is not practically possible. A typical example is a severe depressive condition preceded DLB, which can only be diagnosed with MDD as a prodromal state of the dementia. Additionally, if depression as a BPSD is severe, dementia may be diagnosed with depression. A double-blind randomized controlled trial comparing antidepressants with placebo for depression associated with AD reported no significant differences in changes of depressive symptoms and no expected benefit of antidepressants [101]. When depression in older adults is diagnosed and does not improve or worsen after a certain period of aggressive pharmacotherapy with antidepressants or when new psychiatric symptoms, including cognitive dysfunction and psychotic symptoms, appear during follow-up, it may be necessary to reconsider the possibility of dementia. However, depressive pseudodementia with severe cognitive dysfunction may be diagnosed with dementia. At present, dementia is fundamentally difficult to treat, and the only options for medical intervention are symptomatic treatment of BPSD and nonpharmacological/ pharmacological treatment to control progression. Conversely, depression is a disease in which remission can be expected with the use of antidepressants and nonpharmacological therapies, although the response rate is insufficient in some areas. In addition, antidementia drugs are naturally ineffective for depression, and their side effects may exacerbate anxiety and irritability. Therefore, there is a major problem in continuing to treat depression with the diagnosis of dementia. Even in cases where dementia has been diagnosed, if there is no progression of cognitive dysfunction over time, it is necessary to consider changing the diagnosis. In particular, if the patient has severe depressive symptoms, clinicians need to consider changing the diagnosis to depression, discontinuing the prescription of antidementia drugs, or changing the treatment to depression.

## Burden of and responses to aging and dementia

In the very old, some cancers result in a close to natural death. Dissemination of this fact may help reduce anxiety about cancer and contribute to the withholding of excessive treatment. When cancer is associated with dementia, the question of how far to go with treatment is always a vexing question. A patient with dementia who was admitted to the hospital because of decreased appetite had terminal stomach cancer. After hospitalization, the patient lived in bed but died quietly in two months without any complaints of pain or anguish-like expressions. As a clinician, you may often experience that a patient suffering from severe cancer pain can be averted by dementia [102]. This may seem like one of the benefits of dementia. However, dementia might result in the deterioration of verbal and non-verbal communication, which only prevents patients from effectively communicating the pain they feel. Although patients with dementia can control such pain, they may have pain that is not recognized by the people around them, and there are many such patients. Therefore, based on this factor, it does not mean that dementia has a positive aspect. Considering the enormous burden that dementia places on patients, caregivers, healthcare cost, and systems worldwide, it might be argued that there is nothing positive about this disease [103-105].

In the treatment of depression associated with AD, it is important to listen not only to the patient but also to the caregivers who usually live with the patient and to respond appropriately without overlooking serious depressive symptoms such as self-neglect and thoughts of death. AD patients are prone to feelings of self-doubt and helplessness because they can no longer do what they used to be able to do, and it is desirable for those around them to treat them in a supportive manner. On the other hand, caregivers may be overwhelmed with not only core symptoms such as amnesia but also behavioral and psychological symptoms, which may lead to depression and resentment toward the patient, which may damage the relationship. In such cases, not only a supportive attitude toward the patient but also social support, such as disease education for caregivers and application for long-term care insurance, are effective. There is also a report that exercise therapy is effective, and when exercise therapy was combined with disease education for caregivers, not only physical improvements but also depressive symptom improvements were observed [106]. In a recent study, persistent loneliness was reported to increase the risk of developing dementia (hazard ratio 1.91, 95% CI 1.25-2.90, P<0.01), and supporting social interaction for older adults who tend to be isolated may be one of the effective ways to prevent dementia [107].

Antidepressants remain the mainstay of pharmacotherapy in AD with MDD. This is because antidepressants are effective in a certain number of patients, and alternative treatments other than antidepressants have not yet been identified. On the other hand, the results of large clinical trials and meta-analyses have not shown a clear benefit of antidepressants for depression comorbid with AD [101, 108]. However, these studies do not completely deny the efficacy of antidepressants. Antidepressants may be a reasonable choice for those who still have moderate or severe depressive symptoms after nonpharmacological approaches. While selecting antidepressants, selective serotonin reuptake inhibitors (SSRIs) are the first choice. Long-term treatment with SSRIs has been shown to reduce the risk of AD in depressed patients [109]. However, SSRIs may exacerbate apathy by inducing a decrease in spontaneity, and careful follow-up is necessary when they are used. A recent retrospective study revealed that among 119 outpatients with psychiatric disorders, both the mean apathy scores and the percentage of patients with clinically significant apathy were significantly higher in the SSRIs-treated group than in the non-SSRIs-treated group [110]. With regard to pharmacotherapy, antidepressants should be administered with caution, not only because of their side effects and tolerability but also because of the risk of worsening apathy. On the other hand, in animal experiments, the chronic administration of SSRIs restored stress-induced changes in hippocampal neurogenesis, suppressed the apoptosis of primary hippocampal neurons, and increased BDNF expression [111-115]. Furthermore, in humans, treatment with SSRIs has been suggestive of the possibility of improving the cognitive function and daily life in patients with MCI and AD [116-119]; however, there are only a few studies that have examined the potential of SSRI treatment in reducing the progression from normal cognitive status to MCI or AD. In a prospective longitudinal cohort study of patients with non-depression, long-term treatment (>4 years) with SSRIs in patients with MCI and a history of depression has been found to significantly delay the progression to AD by approximately 3 years; thus, long-term treatment with SSRIs might be capable of slowing the progression from MCI to AD [120]. Therefore, long-term continuous treatment with antidepressants such as SSRIs may facilitate neurogenesis in the human hippocampus, reduce the incidence risk of dementia in people with normal cognitive function, and delay the transition to dementia in patients with MCI.

There is no one who can live without experiencing the phenomenon of aging. Even those who have been fortunate enough to spend their youth, middle age, and early old age free from disease will eventually experience physical problems. In other words, older adults are forced to be aware of their bodies, and psychogenic physical symptoms are likely to appear. In our daily practice, we often encounter patients who complain of physical disorders that seem to be caused by psychological factors. The most important thing for us, as therapists, to do for (very) older patients with so-called psychogenic symptoms is to accept their symptoms in a supportive manner while conducting tests in small doses, minimizing the use of medications, and looking for ways to distract them from their symptoms in their daily lives. A recent systematic review also reported that nonpharmacological interventions are more effective than pharmacological interventions in reducing symptoms of depression in dementia patients without MDD [121]. The best thing to do is to encourage these older adults to immerse themselves in whatever interests them, and if they have outstanding abilities, it is best to help them exercise those abilities. Additionally, since nursing care services are readily available for older adults, it would be a good idea to encourage them to use those services. There are many older adults who are uncomfortable in large groups. It may be considered medical treatment to work with the family or care manager to find alternative measures that take into account the patient's life history.

## Conclusion

It is important to recognize that depression and dementia may share common symptoms and to appropriately differentiate depressed patients who are suspected of having dementia. However, there are cases in which it is difficult to differentiate between the two, and depending on the course of the disease, it may be necessary to change the diagnosis. Regarding psychogenic symptoms seen in older adults, it is not advisable to confront the symptoms but rather to accept the patient and then seek help from others, including individuals familiar with the aging process, those providing nursing care insurance services, and family members. In addition, the aging process is not only a negative feature. It is necessary to consider aging as a change that allows us to become more flexible and natural; therefore, the positive aspects of aging should be considered.
